# The Direct Effect of Magnetic Tape^®^ on Pain and Lower-Extremity Blood Flow in Subjects with Low-Back Pain: A Randomized Clinical Trial

**DOI:** 10.3390/s21196517

**Published:** 2021-09-29

**Authors:** Francisco Selva-Sarzo, Samuel Fernández-Carnero, Rob Sillevis, Héctor Hernández-Garcés, Josep-Carles Benitez-Martinez, Juan-Nicolás Cuenca-Zaldívar

**Affiliations:** 1 Department of Physiotherapy, University of Valencia, 46010 Valencia, Spain; info@franciscoselva.com (F.S.-S.); josep.benitez@uv.es (J.-C.B.-M.); 2 Physiotherapy and Nursing Department, University of Alcala, 28801 Alcalá de Henares, Spain; 3 Department of Rehabilitation Sciences, Florida Gulf Coast University, Fort Myers, FL 33965, USA; rsillevis@fgcu.edu; 4 Intensive Care Service, University Hospital Doctor Peset, 46017 Valencia, Spain; hektorhernandez84@gmail.com; 5 Rehabilitation Service, Guadarrama Hospital, 28440 Madrid, Spain; nicolas.cuenca@salud.madrid.org; 6 Research Group in Nursing and Health Café, Puerta de Hierro Health Research Institute-Segovia de Arana (IDIPHISA), 28222 Madrid, Spain

**Keywords:** Magnetic Tape, low-back pain, power pulsed Doppler, pressure pain threshold

## Abstract

Low-back pain has a high impact on the world population, and solutions are in demand. The behavior of specific physiological processes has been modified using magnetic fields, whether for pain relief, bone consolidation, or improvement of vascularization. The use of tape with magnetic properties could help in these cases. A double-blind randomized clinical trial was designed to use Magnetic Tape^®^ versus placebo Kinesio tape. Blood flow variables were evaluated using pulsed power Doppler ultrasound. Resistance index, pulsatility index, systolic velocity, and diastolic velocity were measured. The pressure pain threshold was measured using algometry in 22 subjects. The results reveal significant differences between the groups for the pulsation index variable (8.06 [5.16, 20.16] in Magnetic Tape^®^ versus 5.50 [4.56, 6.64] in Kinesio tape) and lower (0.98 [0.92, 1.02] for Magnetic Tape^®^ versus 0.99 [0.95, 1.01] for Kinesio tape) in the resistance index variable. The pressure pain threshold variable presented significant differences at multiple levels. The application of Magnetic Tape^®^ causes immediate effects on blood flow and pain and could be a technique of choice for pain modulation. Further studies would be necessary.

## 1. Introduction

Chronic low-back pain (CLBP) is one of the main reasons for loss of function, resulting in job absenteeism and decreased quality of life [1,2]. Chronic low-back pain (CLBP) is defined as mechanical musculoskeletal pain in the lower back that has no known cause and lasts for more than 12 weeks [3]. The probability of developing low-back pain is high, with reported lifetime prevalence rates greater than 20% [4]. Research has shown several conditions associated with CLBP [5], such as sex—it is common in women—obesity, smoking, and aging. Commonly pain relief is one of the primary goals in the rehabilitation of patients with low-back pain [6]. Conservative interventions managing low-back pain typically include physical therapy and medication management [7]. Additionally, it has been demonstrated that therapeutic exercise such as the McKenzie method, stabilization exercises, and strength-building exercises [8] can be beneficial.

At the cellular level, endogenous ion fluctuations are important for the regulation of cell activity. Biologically, bioelectrical signals provide pathways to restore normal functioning and homeostasis after injury [9]. It has been demonstrated that magnetic fields positively influence human tissue and result in several therapeutic effects, such as pain relief, bone regeneration, muscle regeneration, improved nerve functioning, and improvement in tissue vascularization [10,11,12,13,14,15,16,17,18]. Additionally, animal studies have identified increased vascularization in rabbits when exposed to static magnetic fields (SMFs) [16].

SMF therapy has been used for centuries to control pain, but the mechanism by which it reduces pain is unclear. One theory proposes that nociceptive C fibers have a lower threshold potential and that magnetic fields selectively attenuate neuronal depolarization by changing the resting membrane potential [10,19]. A second theory suggests that magnetic fields promote increased blood flow through the skin, subcutaneous tissue, muscle tissue, and ligament tissues [20]. A third theory suggests that SMFs affect the kinetics of ion binding in cellular macromolecules directly, which modulates the release of cytokines and other factors [21,22].

Since the 1990s, there has been an expansion in efforts to develop various medical applications using SMF therapy and, specifically, magnetic nanoparticles (MNs). External magnetic fields interact with magnetic nanoparticles and thus could have a direct effect on tissues, cells, or biomolecules [23]. From the different MNs that have been developed, those that present a rapid change of magnetic state with the application of an external magnetic field are usually desired [12]. Due to their small dimensions, MNs do not exhibit any magnetization unless in contact with an external magnetic field [23]. The clinical application of magnetic nanomaterials is due to their biocompatibility, versatility, and being minimally toxic and highly sensitive even to small external particles, creating a wide range of uses [23].

Magnetic nanoparticles are attracting increased attention due to their potential to improve conventional therapeutic procedures and clinical diagnostics, thus providing novel biomedicine approaches [12]. Magnetic nanoparticles can be designed for the prevention, diagnosis, and treatment of diseases [12]. The result of the review carried out by McKay et al. [16] indicates that nearly half of the cited experiments (10 of 27 studies) reported a vasodilatory effect due to magnetic fields (MFs). Magnetic nanoparticles are emerging as an essential class of biomedical functional nanomaterials in areas such as hyperthermia. Combining magnetic particles with polymeric biomaterials has shown great potential for tissue repair, such as bone, muscle, nerve, and cardiac tissue regeneration [8]. The application of magnetic hyperthermia (MH) makes this technique a promising tool for cancer treatments and is currently one of the more intense areas of nanotechnology research in the biomedical field [12].

Based on current evidence, it could be concluded that magnetotherapy could provide a non-invasive, safe, and easy method to treat pain [24]. Furthermore, magnetotherapy might offer the potential to reduce spending on medical care for the management of chronic musculoskeletal disorders [25]. Kinesiology tape (KT) has been used to influence vascularization and blood flow using various application methodologies. If KT is applied with or without tension, it does not have local effects on resistance indices, blood flow, circumference, and volume [26]. There has been no report of increased paraspinal blood flow nor a decrease in the perceived pain in the spine [27].

Magnetic Tape^®^ is an adhesive elastic tape that incorporates magnetic nanoparticles without the ability to create magnetic fields until it comes into contact with electromagnetic fields such as those generated by living beings (see Supplementary Material Video 1). The primary aim of this study was to investigate the effect of Magnetic Tape^®^ with magnetic nanoparticles (MT) applied to the lumbosacral area in subjects with low-back pain on the vascularization of the lower limbs. The secondary aim was to determine if the Magnetic Tape^®^ had an immediate effect on pain with posterior to anterior pressure applied to the spinous processes of the spine.

## 2. Materials and Methods

**Figure 1:**
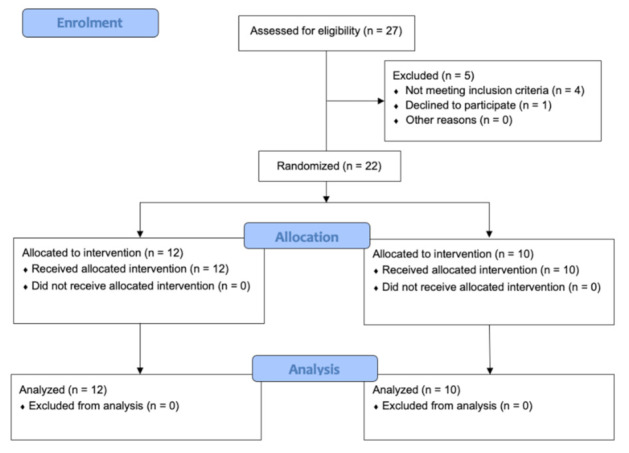
CONSORT flow diagram.

A randomized double-blinded clinical trial was designed. For this pilot study, a convenience sample of patients with low-back pain was selected from the private practice of the faculty of Physiotherapy of the University of Valencia (Spain). The ethics committee from the University of Valencia approved this study (Nº 1551975), and it was registered on clinicaltrials.gov (NCT04836468). The recommendations of the “Consolidated Standards of Reporting Trials” (CONSORT) were followed [28] (Figure 1).

The subjects for this study were recruited based on the study inclusion and exclusion criteria. The inclusion criteria consisted of: subjects with low-back pain, aged between 18 and 65 years, living in Valencia, diagnosed by a medical doctor, and able to read Spanish so that informed consent could be given. The exclusion criteria were not having the age requirement, having conditions that would be a contraindication for the adhesive tape such as allergies, being pregnant, having a pacemaker, any contraindication of electromagnetic fields, neurological diseases, or taking any medication that may interact with magnetic fields. If the subjects met the study criteria, they provided written consent to participate in this study. At that point, demographics and preintervention data were collected.

**Figure 2:**
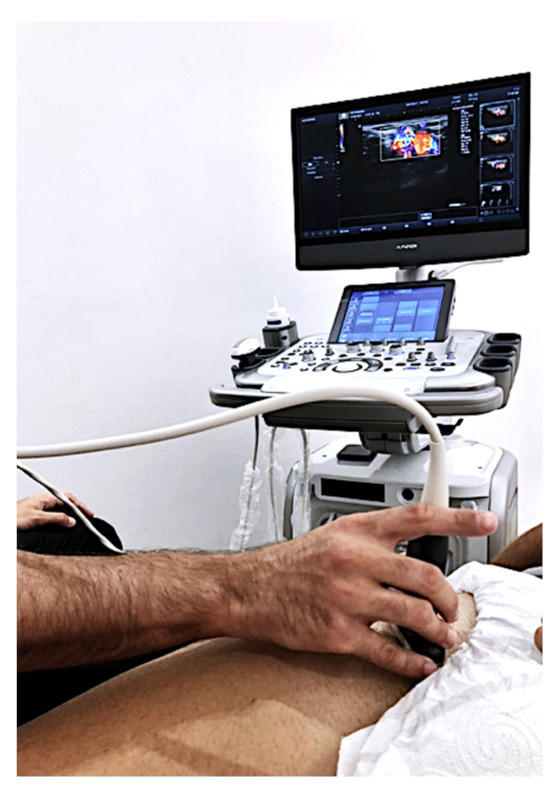
Pulsed Doppler exam.

To measure blood flow in each lower extremity of the subjects, the Alpinion E-CUBE 12 ultrasound device (Alpinion Medical Systems Co., Ltd., Seoul, Korea) with an L3-12H linear array was used (64 mm footprint). Doppler ultrasound software was installed. A sonographer with more than ten years’ experience measured, with pulsed power Doppler ultrasound, blood flow in the femoral artery of the subjects in the supine position. Systolic speed (SM), diastolic speed (SD), the ratio between both (SD), pulsation index (PI), and arterial resistance index (IR) were measured (Figure 2). Another researcher measured the pressure pain threshold (PPT) using a Wagner Force Dial FDK 20 algometer with a 1cm^2^ footprint following the protocol published in previous studies [29]. The PPT was evaluated with the subjects in the prone position.

**Figure 3:**
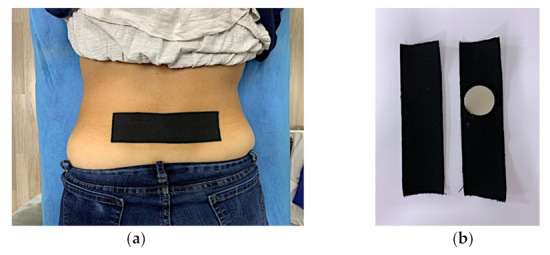
Magnetic Tape^®^ and Kinesio tape application. Tape application in lumbar region (**a**) and tape preparation, only neodymium magnet allow identification (**b**).

After baseline blood flow and PPT measures were obtained, the subjects were randomized using sealed envelopes and then were taped, by a third researcher, with either the Magnetic Tape^®^ (MT) or placebo (KT). During testing, the researchers and subjects were blinded for the type of tape application, patient allocation, or patient identification. The tape was applied transversely relative to the spinous processes of L4 and L5. There was 0% elongation of the tape, thus not creating any tension both in the MT and KT tape application. The MT and KT tapes were alike in appearance (Figure 3). Following the tape application, the pulsed power Doppler ultrasound blood flow and PPT measures were repeated. 

### Statistical Analysis

For the statistical analysis, the R Ver. 3.5.1. (R Foundation for Statistical Computing, Institute for Statistics and Mathematics, Welthandelsplatz 1, 1020 Vienna, Austria) was used. The level of significance was established at *p* < 0.05. The distribution of quantitative variables of each test was tested with the Shapiro–Wilk test. This analysis demonstrated that the variables were not normally distributed for both groups. Quantitative variables were described with median and interquartile range and qualitative variables with absolute and relative frequencies. Due to the lack of normality of the data and the small sample size [30], an exact permutation test was applied to the final values of the outcome variables between groups. The effect size was defined with the non-parametric r statistic, as 0.1–0.4 (small), 0.4–0.6 (moderate), and >0.6 (large).

## 3. Results

A total of 22 subjects were sampled by randomization: 12 (*n* = 12) received the Magnetic Tape^®^ KT, and the remaining subjects received the placebo KT (n = 10). The demographic distribution of the variables for blood flow in systolic pressure (SM), diastolic pressure (SD), the ratio between both (SD), pulsation index (PI), and arterial resistance index (IR) can be found in Table 1. There were no significant differences between baseline groups.

Following the application of either tape, the subject’s pain perception was measured utilizing the pressure pain threshold. A significant difference at multiple vertebrae levels was found. The distribution of quantitative variables of each test was tested with the Shapiro–Wilk test. This analysis demonstrated that the variables were not normally distributed in both groups. For this reason, non-parametric statistics were used to evaluate the data.

A hypothesis test was performed to check whether the application of the Magnetic Tape^®^ represents a significant change in either the PPT and/or arterial flow (Table 2 and Table 3).

### 3.1. Non-Parametrics Analysis: Mann–Whitney U Test

We applied the Mann–Whitney U test between groups with the post-treatment values of all the variables, verifying that there were no significant differences (Table 3).

### 3.2. Permutation Test

We applied a permutation test, both with the Monte Carlo simulation and exact, between groups with the post-treatment values of all variables. With the Monte Carlo simulation, significant differences were observed between groups in the PI variable, while with the exact permutation test, the differences occurred in the PI and IR variables (Table 4).

### 3.3. Mann–Whitney U Test with the Pre-Treatment Difference

We applied the Mann–Whitney U test between groups with the pre-treatment difference of all variables. There are significant differences between the two groups in the PI variable (Table 5).

### 3.4. Permutation Test with Pre-Treatment Difference

We applied a permutation test, both with the Monte Carlo simulation and exact, between groups with the pre-treatment difference of all variables. With the Monte Carlo simulation, significant differences between groups are observed in the PI variable, while with the exact permutation test, the differences occurred in the PI and IR variables.

### 3.5. Non-Parametric Ancova

A non-parametric ANCOVA was calculated for each variable using permutations, with the post-treatment result as the dependent variable, the group as the variable of interest, and the pre-treatment variable as the baseline covariate. Significant differences between the two groups were found in the PI variable.

### 3.6. Boostrap Linear Mixed Model

A bootstrap mixed linear model with a random intercept was calculated for each variable, with the post-treatment result as the dependent variable, the group as the variable of interest, the pre-treatment variable as the baseline covariate (fixed effects), and the subjects as random effects. Significant differences between the two groups were observed in the IR variable (Table 6).

We evaluated the model with the significant PI variable with the backward stepwise method, starting with fixed effects saturated with the pre-treatment Group–SM interaction and ending with the random-effects model, only with the intercept. The ANOVA table shows significant differences between the Group*SM versus Group+SM (*p* = 0.006) and Group+SM versus Group (*p* < 0.001) models; however, the more complex model is non-significant, and thus we chose the initial Group+SM model that adequately explains the variability of the data and is significant in the group (Table 7).

The linear mixed model shows how the final values in the PI variable in the placebo tape group are −4.255 ± 2.479 times lower than in the Magnetic Tape^®^ group; this difference is significant (regression coefficients (95%CI) = −9.694, −0.875) with a large and significant effect size (R^2^ = 0.93, 95%CI (0.883, 0.966)) (Table 8).

### 3.7. Pain Analysis

We applied an exact permutation test between groups with all variables. The pain was measured using the pressure pain threshold and showed significant differences at multiple vertebrae levels (Table 9).

Furthermore, there were significant differences between both groups in the PI (Z = 1.349, *p* < 0.001) and IR (Z = 0.54, *p* < 0.001) variables, with a small and significant effect size. In both cases, the final values were higher in the Magnetic Tape^®^ group (8.06 [5.16, 20.16] versus (5.50 [4.56, 6.64]) in the PI variable and lower (0.98 [0.92, 1.02]) versus (0.99 [0.95, 1.01]) in the IR variable. In the Magnetic Tape^®^ group, there was an increase in the values of the PI variable (from 5.11 [4.14, 6.11] to 8.06 [5.16, 20.16]) with a slight increase in the IR variable (from 0.95 [0.87, 1.04] to 0.98 [0.92, 1.02]). In addition, in the placebo tape group, there was a lower increase in the values of the PI variable (from 4.14 [3.85, 6.04] to 5.50 [4.56, 6.64]) with an increment similar to the Magnetic Tape^®^ group in the IR variable (from 0.95 [0.88, 0.99] to 0.99 [0.95, 1.01]) (Table 2). 

## 4. Discussion

The primary aim of this study was to investigate whether the effect of a tape with magnetic nanoparticles applied to the lumbosacral area in subjects with low-back pain has a systemic effect on the vascularization of the lower limbs. The secondary aim was to determine if the Magnetic Tape^®^ had an immediate effect on pain with posterior to anterior pressure applied to the spinous processes of the spine.

The results of this study reveal the modulation of blood flow, evaluated with pulsed Doppler ultrasound at the femoral artery. The modifying variables of the arterial resistance index and the pulsatility index were three times more than in the placebo group. Additionally, there was a decrease in the level of PPT at various vertebral levels.

Our research findings are in line with the results of previous studies of McKay et al. [11]. Results of Monfrecola et al. [31] demonstrate improved cutaneous blood flow by 62%. In the same review, a study with mice was incorporated where the speed of the blood flow increased in a range of 15% to 45%. In our study, the application of SMFs produced an increase in blood flow between 20% and 40%, which concurs with the findings of Monfrecola et al. [31].

Schuhfried et al. [32] studied the effect of time-varying SMFs on microcirculation and the alteration of temperature in human feet. Low-frequency fields were used, causing an increase in blood velocity in a range of 20% to 45% after exposure. The pulsed power Doppler measurements observed in very light exercise do improve the mean blood velocity (MBV) of the femoral artery from 10 cm/sec to 20 cm/s [33]. Similar results were found after a slight quadriceps contraction, causing a rapid increase in the MBV of the femoral artery from 10.1 cm/s to 28.1 cm/s [34]. The findings of our study are comparable to these results, which indicates that the application of Magnetic Tape^®^ could help improve vascularization. Any variation in the diameter of the small vessels will more easily affect the diastolic velocity. In contrast, variations in the large vessels reflect a more significant variation in systolic velocities with increased blood flow velocity [35]. The sympathetic nervous system stimulation induced by the Magnetic Tape^®^ could be responsible for the observed blood flow changes.

Gossling HR et al. [13] demonstrated in 1992 that the activation of body magnetic fields in different traumatic pathologies could be as effective as surgical interventions. Li Y et al. [14] reached the same conclusions in 2020. Both Gossling et al. and Li et al. concluded that the treatment of non-healing bone fractures with electromagnetic fields has proven more successful than the traditional approach. Given the costs and potential dangers of surgery, magnetic field therapy should be considered an effective alternative. Additionally, for healing to occur, it is necessary to improve vascularization. Our study also demonstrated that NMs reduce pain.

Current trends in biology and medicine research using MNs are evaluating the effects on tissues, cells, and biomolecules [12]. MNs can be designed for the prevention, diagnosis, and treatment of disease [12]. Magnetic compounds activated through external magnetic fields have been shown to further enhance the biological properties of cells [11]. They can stimulate endothelial cell proliferation, promoting osteogenesis for bone repair in vivo [11]. One of the most intense areas of nanotechnology research in the biomedical field is hyperthermia due to increased vascularization [12]. The nanoparticles used in this study were activated by external magnetic fields of the body [23]. Our study implies that the Magnetic Tape^®^ nanoparticles created a rapid change in the epidermis’s magnetic state affecting the local blood flow.

The effect of the Magnetic Tape^®^ on pain is comparable with Brown et al. [10] in which chronic low-back pain improved by 40% after using 500 Gauss active magnets on trigger points, while the use of placebo magnets produced a 3% worsening of symptoms. The magnetic field acts as the vehicle to induce ion flow and does not stimulate the nerve tissue itself [36]. However, once the ion flow is created in the epidermal cells, the mechanism of electrical and magnetic stimulation at the neural level is the same, producing the depolarization of the axon and the initiation of the action potential [36]. Additionally, epidermal cells, especially the Langerhans cells, on which ion flow is induced, act on the lymphatic system and are innervated by the sympathetic autonomic nervous system [37]. A recent publication by Chao et al. 2021 [38] related epidermal dysfunctions to modifications in the thalamus; this implies a centralized effect. We also postulate that it is a priority due to its ectodermal embryological origin.

We agree with Brown et al. [10] that the skin is a part of the mechanism by which SMFs act on our body in a systematic way. The hypothesis of the mechanism of action of Magnetic Tape^®^ is that it acts superficially on epidermal cells. Our results demonstrate physiological baseline state changes. Future research is needed to explore the possible benefits for other conditions and locations using Magnetic Tape^®^.

Finally, we would like to mention that clinicians have used Kinesiology tape or Kinesio to influence vascularization and blood flow in the area of the tape application [25,26,27]. However, the effects of kinesiology tape (KT) on vascular flow and pain have not been demonstrated. This concurs with previous systematic reviews [39,40], revealing no relation between KT and pain relief.

## 5. Limitations

To our knowledge, no previous studies evaluated the effects of magnetic nanoparticles in a tape on an epidermis. Our study only evaluated the immediate effects of the Magnetic Tape^®^ on pain and vascularization. Therefore, no middle and long-term effect can be inferred. Future research is needed to evaluate long-term effects and further explore the effects of the Magnetic Tape^®^ on microcirculation. Additionally, the effects of Magnetic Tape^®^ on the autonomic nervous system should be explored. 

## 6. Conclusions

The application of Magnetic Tape^®^ in subjects with low-back pain resulted in immediate and significant pain reduction. There was an immediate increase in blood flow parameters after the application of Magnetic Tape^®^. The reduction of pain and the improvement of the analyzed blood flow parameters indicate that Magnetic Tape^®^ could be used to manage pain. Magnetic Tape^®^ provides a non-invasive, safe, and simple method to manage perceived pain. Further studies are needed to explore further how magnetic nanoparticles affect pain perception.

## Figures and Tables

**Table 1:** Participant baseline demographic and clinical characteristics.

Demographics and Variables	Gender	Magnetic Tape^®^ Group	Placebo Tape Group	*p* Value ^a^
n		12	10	
Gender, n (%)	Female	6 (50.0)	4 (40.0)	0.969
	Male	6 (50.0)	6 (60.0)	
SM		33.09 [27.52, 40.62]	29.45 [22.86, 37.66]	0.356
DF		3.07 [1.67, 4.47]	2.09 [0.47, 4.04]	0.448
SD		7.65 [4.85, 14.96]	8.96 [6.29, 21.93]	0.575
PI		5.11 [4.14, 6.11]	4.14 [3.85, 6.04]	0.644
IR		0.95 [0.87, 1.04]	0.95 [0.88, 0.99]	0.843

Data expressed with median [interquartile range] or with absolute and relative values (%). Systolic pressure (SM), diastolic pressure (SD), ratio between both (SD), pulsation index (PI), and arterial resistance index (IR). ^a^ Significant if *p* < 0.05.

**Table 2:** Outcome variable results.

Variables	Magnetic Tape^®^ Group	Placebo Tape Group	Difference (95%CI)	*p* Value ^a^	r (95%CI)
** SM **	22.57 [20.21, 27.45]	25.86 [20.29, 32.41]	−3.29 (−10.42, 3.77)	1	0.141 (0, 0.571)
** DF **	1.31 [0.45, 1.92]	0.77 [0.20, 2.16]	0.535 (−1.21, 1.31)	1	0.127 (0.018, 0.515)
** SD **	17.04 [11.56, 57.00]	53.83 [10.75, 104.25]	−36.79 (−77.14, 11)	1	0.155 (0.007, 0.499)
** PI **	8.06 [5.16, 20.16]	5.50 [4.56, 6.64]	2.565 (−0.86, 14.7)	<0.001	0.323 (0.023, 0.668)
** IR **	0.98 [0.92, 1.02]	0.99 [0.95, 1.01]	−0.005 (−0.07, 0.1)	<0.001	0.007 (0.011, 0.519)

Data expressed with median [interquartile range]. R: non-parametric effect size. 95%CI: 95% confidence interval. ^a^ significant if *p* < 0.05. Systolic pressure (SM), diastolic pressure (SD), ratio between both (SD), pulsation index (PI), and arterial resistance index (IR).

**Table 3:** Mann-Whitney U test calculations.

Variables	*p* Value ^a^
**SM**	0.539
**DF**	0.571
**SD**	0.497
**PI**	0.14
**IR**	0.987

Systolic pressure (SM), diastolic pressure (SD), ratio between both (SD), pulsation index (PI), and arterial resistance index (IR). ^a^ Significant if *p* < 0.05.

**Table 4:** Permutation test.

Monte Carlo Simulation		Exact Permutation	
Variables	*p* Value ^a^	Variables	*p* Value ^a^
** SM **	0.928	SM	1
** DF **	0.72	DF	1
** SD **	0.653	SD	1
** PI **	0.015	PI	<0.001
** IR **	0.359	IR	<0.001

Systolic pressure (SM), diastolic pressure (SD), ratio between both (SD), pulsation index (PI), and arterial resistance index (IR). ^a^ Significant if *p* < 0.05.

**Table 5:** Mann–Whitney U test.

Variables	Group *p* Value ^a^
SM	0.157
DF	0.784
SD	0.420
PI	0.005
IR	0.706

Systolic pressure (SM), diastolic pressure (SD), ratio between both (SD), pulsation index (PI), and arterial resistance index (IR). ^a^ significant if *p* < 0.05.

**Table 6:** Bootstrap mixed linear model.

Variables	95%CI Bootstrap Group Regression Coefficients ^a^
**SM**	−2.739, 9.42
**DF**	−1.339, 1.799
**SD**	−19.34, 64.592
**PI**	−9.694, −0.875
**IR**	−0.092, 0.049

Systolic pressure (SM), diastolic pressure (SD), ratio between both (SD), pulsation index (PI), and arterial resistance index (IR). 95%CI: 95% confidence interval. ^a^ significant if it does not contain the zero value.

**Table 7:** Evaluation of the model with the PI variable.

	AIC	BIC	Log Likelihood-Ratio Test	*p* Value ^a^
Group*SM vs. Group+SM	145.528	150.983	7.625	0.006
Group+SM vs. Group	202.227	206.592	58.699	<0.001
Group vs. Intercept	202.221	205.494	1.994	0.158

Systolic pressure (SM). ^a^ significant if *p* < 0.05.

**Table 8:** Linear mixed model.

Coefficients	Standard Error	Degrees of Freedom	t Value	95%CI Bootstrap Regression Coefficients ^a^	
Intercept	−1.634	2.055	19	−0.795	−5.557, 3.062
Group (placebo)	−4.255	2.479	19	−1.716	−9.694, −0.875

^a^ significant if it does not contain the zero value.

**Table 9:** Exact permutation test: pressure pain threshold.

Vertebral Level	Magnetic Tape^®^ Group	Placebo Tape Group	*p* Value ^a^
L1 postreatment first time	6.3 ± 1.149	4.32 ± 0.955	<0.001
L1 postreatment second time	6.333 ± 1.15	4.3 ± 0.957	<0.001
L2 postreatment first time	6.35 ± 0.967	4.62 ± 0.55	<0.001
L2 postreatment second time	6.3 ± 0.844	4.62 ± 0.55	<0.001
L3 postreatment first time	6.583 ± 0.913	4.38 ± 0.726	<0.001
L3 postreatment second time	6.633 ± 0.956	4.38 ± 0.76	<0.001
L4 pretreatment first time	3.85 ± 0.602	3.68 ± 0.502	<0.001
L4 pretreatment second time	3.883 ± 0.688	3.68 ± 0.563	<0.001
L4 postreatment first time	6.567 ± 0.878	3.64 ± 0.483	<0.001
L4 postreatment second time	6.517 ± 0.868	3.62 ± 0.507	<0.001
L5 postreatment first time	6.133 ± 1.035	3.72 ± 0.676	<0.001
L5 postreatment second time	6.117 ± 1.042	3.68 ± 0.638	<0.001
S1 postreatment first time	6.75 ± 0.92	4.28 ± 0.642	<0.001
S1 postreatment second time	6.783 ± 0.972	4.32 ± 0.642	<0.001
S2 postreatment first time	6.767 ± 0.802	4.52 ± 0.228	<0.001
S2 postreatment second time	6.833 ± 0.812	4.56 ± 0.251	<0.001
S3 postreatment first time	6.8 ± 0.817	4.48 ± 0.295	<0.001
S3 postreatment second time	6.8 ± 0.883	4.54 ± 0.336	<0.001
S4 postreatment first time	6.8 ± 0.81	4.74 ± 0.288	<0.001
S4 postreatment second time	6.833 ± 0.821	4.72 ± 0.277	<0.001

^a^ showed only significant differences set at *p* < 0.05.

## Supplementary Materials

The following are available online at https://www.mdpi.com/article/10.3390/s21196517/s1, Video S1: Magnetic Tape test.
